# Gum-Chewing

**Published:** 1885-07

**Authors:** 


### GUM-CHEWING.

For a proper comprehension of Dr. Wright's article in this number, the reader should again peruse the discussion in the Mississippi Valley Association, reported in the May number of this Journal. There is a grave question involved in this matter, although Dr. Wright considers it in a facetious manner. With the article he sends a characteristic list of titles, and we present them all, that the reader may select for himself :

"The Fathers eat Sour-Crout and the teeth of the Children are set on edge ! ! "

"A short visit to the Third and Fourth Generations ! ! ! "

"A new variety of jawbone, *a la* Sampson ! ! ! ! "

"The Chewsharp of the Mississippi Valley Dental Society ! ! ! ! ! "

"Transmission of acquired characteristics through a carefully cultivated system of Gum-Chewing ! ! ! ! ! ! "

